# METTL3 Promotes the Resistance of Glioma to Temozolomide *via* Increasing MGMT and ANPG in a m^6^A Dependent Manner

**DOI:** 10.3389/fonc.2021.702983

**Published:** 2021-07-15

**Authors:** Jia Shi, Gang Chen, Xuchen Dong, Haoran Li, Suwen Li, Shan Cheng, Yongdong Li, Liping Wang, Jiaqi Yuan, Zhiyuan Qian, Jun Dong

**Affiliations:** ^1^ Department of Neurosurgery, The Second Affiliated Hospital of Soochow University, Suzhou, China; ^2^ Department of Neurosurgery, The Third Affiliated Hospital of Soochow University, Changzhou, China; ^3^ Department of Neurosurgery, Zhuhai People’s Hospital, Zhuhai Hospital Affiliated With Jinan University, Zhuhai, China

**Keywords:** glioblastoma, temozolomide, resistance, N6-methyladenosine (m 6 A), METTL3

## Abstract

Acquired chemoresistance is a major limiting factor in the clinical treatment of glioblastoma (GBM). However, the mechanism by which GBM acquires therapeutic resistance remains unclear. Here, we aimed to investigate whether METTL3-mediated N6-methyladenosine (m^6^A) modification contributes to the temozolomide (TMZ) resistance in GBM. We demonstrated that METTL3 METTL3-mediated m^6^A modification were significantly elevated in TMZ-resistant GBM cells. Functionally, METTL3 overexpression impaired the TMZ-sensitivity of GBM cells. In contrast, METTL3 silencing or DAA-mediated total methylation inhibition improved the sensitivity of TMZ-resistant GBM cells to TMZ *in vitro* and *in vivo*. Furthermore, we found that two critical DNA repair genes (MGMT and APNG) were m^6^A-modified by METTL3, whereas inhibited by METTL3 silencing or DAA-mediated total methylation inhibition, which is crucial for METTL3-improved TMZ resistance in GBM cells. Collectively, METTL3 acts as a critical promoter of TMZ resistance in glioma and extends the current understanding of m^6^A related signaling, thereby providing new insights into the field of glioma treatment.

## Introduction

Owing to the introduction of temozolomide (TMZ), an alkylation agent, and the use of radiotherapy in combination with TMZ adjuvant therapy, the median survival of patients with glioblastoma multiforme (GBM) was increased from 12.1 months to 14.6 months (1–5). However, the overall clinical efficacy of this regimen remains disappointing, mainly because of inherent or induced resistance to TMZ treatment (6–11). Mostly, TMZ-resistant cell lines highly expressed O^6^-methylguanine-DNA methyltransferase (MGMT) and alkylpurine–DNA–N-glycosylase (ANPG) (12, 13). TMZ methylated 12 kinds of DNA bases at different sites, of which, O^6^-meG was considered the most toxic lesion (14). MGMT repairs O^6^-meG through a suicidal response, thereby becoming resistant to TMZ. On the other hand, ANPG repairs the cytotoxic lesions N^3^-methyladenine and N^7^-methylguanine and contributes to TMZ resistance (12). Therefore, the clinical treatment of this deadly tumor urgently requires a more comprehensive understanding of its progression, mechanisms of resistance, and new therapeutic targets.

In the eukaryotic cells, N6-methyladenosine (m^6^A) is the predominant modification of mRNA and long non-coding RNAs (15). m^6^A is a dynamic and reversible RNA modification in mammalian cells that occurs after transcription by the m^6^A methyltransferase complex, which contains the enzyme subunit methyltransferase-like 3 protein (METTL3) and its co-cofactors methyltransferase-like 14 protein (METTL14) and WT1-associated protein (WTAP) (16). With the deepening understanding of RNA methylation, a number of regulatory factors involved in the regulation of mammalian m^6^A have been identified (17).

The m^6^A modification of the methyltransferase-imprinted RNA prioritizes the recognition and delivery of the reader protein and is cleared by RNA demethylase (18). Therefore, three types of regulators dynamically controlling m^6^A are defined as writers, readers, and erasers (19). Under the control of these three regulatory factors, m^6^A methylation epigenetic regulation of a large number of gene expression plays multiple roles in the regulation of biological processes (20). The acquisition of m^6^A reduces the stability of transcription and mediates the attenuation of target mRNA, suggesting that m^6^A modification is a negative regulator of mRNA translation. Instead, m^6^A deficiency increases the abundance and longevity of transcripts, as well as the overall expression of the protein. m^6^A can also change the structure of RNA, promote the binding of protein regulators, affect mRNA maturation, and regulate gene expression.

It has been reported that m^6^A modification plays a variety of regulatory roles in tumor initiation, progression, and radiation resistance (21, 22). In addition, a growing body of evidences suggests that genetic alterations and dysregulation of m^6^A RNA methylation regulators are closely associated with the malignant progression of a variety of cancers (23). In recent years, increasing evidences have shown that METTL3 plays an important role in cancer as an m^6^A methyltransferase, both as an oncogene and as a tumor suppressor gene. In most cases, METTL3 has been reported as an oncogene that promotes the occurrence and progression of a variety of cancers, including hematopoietic malignancies and solid tumors, by depositing m^6^A modifications on key transcripts (24, 25).

However, the clinicopathological effects of METTL3-mediated RNA m^6^A modification and the related mechanisms of TMZ resistance in glioma have not been elucidated. In this study, we demonstrated that METTL3 acts as a critical promoter of TMZ resistance in glioma. Based on these findings, we provide new insights into the METTL3-mediated modification of m^6^A. We also explored the molecular mechanisms underlying TMZ resistance of glioma by identifying downstream target genes and signals. Therefore, our work extends the current understanding of m^6^A-related signaling and provides new insights into the field of glioma research.

## Materials and Method

### Cell Lines and Cell Culture

Human glioblastoma-derived U87-MG and U251 cell lines were obtained from the American Type Culture Collection (ATCC, Manassas, VA, USA). All cell lines were cultured in DMEM (Gibco, Grand Island, NY, USA) supplemented with 10% FBS (Gibco) and 1% PS (Invitrogen, Carlsbad, CA, USA), and maintained at 37°C and 5% CO_2_ in a humidified atmosphere. TMZ-resistant cell lines were generated by exposure of U87-MG and U251 cells with 200 μM TMZ for over 6 months. The derived resistant cell lines were designated as U87-MG-TMZ resistant and U251-TMZ resistant, respectively. The cell survival ratio and half maximal inhibitory concentration (IC_50_) of TMZ for U87-MG and U251 was evaluated using the CCK-8 assay (**Supplementary Figure 1**).

### Real-Time Quantitative PCR (qRT-PCR)

RNA extraction and real-time fluorescent quantitative PCR (qRT-PCR) were performed as previously described. The relative gene expression of mRNA was calculated by 2^-ΔΔCT^ method. GAPDH was used as an endogenous control to normalize the data.

### Plasmid Transfection

Stable overexpression of METTL3 was achieved by constructing a lentiviral vector (Biospec Technology, Shanghai). In addition, we synthesized shRNA-targeting genes. Transfection of the expression plasmid in glioma cells was performed using Lipofectamine 3000 kit (Invitrogen, Carlsbad, CA, USA) according to the manufacturer’s instructions.

### Western Blot

The cells were directly lysed in 1× SDS-PAGE loading buffer. Protein bands were detected sequentially with primary and HRP-bound secondary antibodies, visualized using a Chemiluminescence Detection Kit (Servicebio, Wuhan, China), and detected with an imaging system (Bio-Rad, USA). Antibodies against METTL3 (AB195352, 1:2000) were obtained from Abcam. GAPDH (60004, 1:5000) antibodies were purchased from Proteintech.

### Total RNA m^6^A Quantification

The total level of m^6^A in the treated glioma cells was determined using the EpiQuik™ m^6^A RNA Methylation Quantitative Kit (Epigentek, USA). Briefly, 200 ng of RNA was added to each well, followed by a mixture of capture and detection antibodies. After several weeks of incubation, the m^6^A content was quantified at 450 nm and calculated according to the standard curve.

### Dot Blot

The mRNA samples were dissolved in a 3-fold volume of RNA incubation buffer, denatured at 65°C for 5 min, and loaded onto an Amersham Hybond-N+membrane (GE Healthcare, USA) mounted on a Bio-Dot device (Bio-Rad, USA). After blocking the membrane with 5% skimmed milk, the specific m^6^A antibody (1:1000, Abcam) was incubated overnight at 4°C. Mouse immunoglobulin G (IgG) was incubated with HRP-conjugated immunoglobulin G (IgG) for 1 h, and imaging was performed using an imaging system (Bio-Rad, USA).

### Methylated RNA Immunoprecipitation (Me-RIP)

Total RNA or poly(A)+mRNA was isolated using the above methods. The purified mRNAs and magnetic bead-antibody complexes were then added to IP buffers and incubated overnight at 4°C, followed by elution with eluent and purification. MGMT and ANPG in RNA were extracted using RT-qPCR.

### Cell Viability Assay

Cell viability was measured after treatment with different concentrations of TMZ (Selleck Chemicals, Houston, TX, USA). After 4 h of normal culture, 10 µL CCK-8 reagent (Dojindo) was added and absorbance at 450 nm was detected using an ultra-multifunctional microplate analyzer (Tecan, Durham, NC, USA). Using GraphPad Prism 9.0 (GraphPad Software, San Diego, CA, USA), the “log (inhibitor) *vs* normalized slope of response variable” method was used to calculate the 50% inhibition concentration (IC_50_) of TMZ.

### Colony Formation Experiments

Glioma cells were seeded in a 6-well culture plate containing 500 cells per well for 14 d. The colonies were washed with PBS and fixed with 4% paraformaldehyde. Photographs were taken using a microscope (Olympus, Ishikawa, Japan).

### Subcutaneous Glioma Xenograft Model

All experiments involving mice were conducted in accordance with the ethical standards of the animal care and use committee of the third hospital affiliated to Soochow University and the NIH guidelines for the care and use of laboratory animals. To establish the xenograft model of glioma in mice, 1×10^7^ human U87-MG-TMZ cells (sh-Con, sh-METTL3, or normal U87-MG-TMZ) were subcutaneously inoculated into the right posterior limb of BALB/c nude mice (6-week-old, female) in 80 μL PBS. Tumor volume was measured with calipers every 5 d. After approximately 30 d, all mice were euthanized, and the tumor masses were removed, weighed, and embedded for further pathological study.

### Statistical Analyses

SPSS 21.0 statistical software (IBM Corp. Armonk, NY) was used for statistical analyses, and statistical significance was set at *P*<0.05. Data are expressed as mean ± standard deviation. Multiple sets of data were evaluated using one-way analysis of variance (ANOVA), and multiple comparisons were performed using Tukey’s post-hoc test. Time-based multiple comparisons were tested by repeated analysis of variance and the Bonferroni post facto test.

## Results

### METTL3 Mediated m^6^A Is Elevated in the TMZ-Resistant GBM Cells

**Figure 1:**
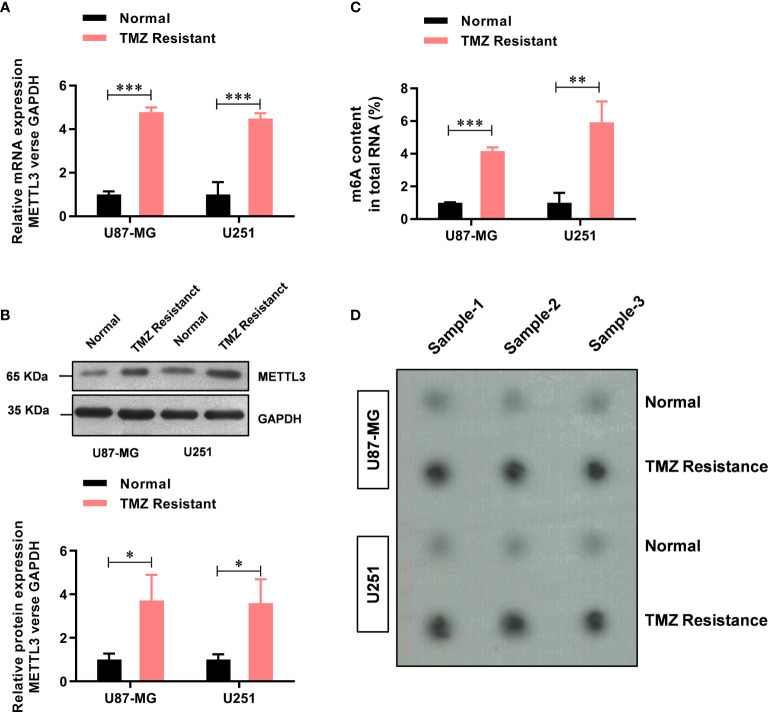
METTL3-mediated m^6^A is elevated in the TMZ-resistant GBM cells. **(A)** The mRNA level of METTL3 in TMZ sensitive and resistant U87-MG/U251 cells was analyzed by real-time PCR. **(B)** The protein level of METTL3 in TMZ sensitive and resistant U87-MG/U251 cells was analyzed by western blot. **(C)** The colorimetric m^6^A quantification assay was used to examine the total m^6^A levels in the TMZ sensitive cells and the resistant U87-MG/U251 cells. **(D)** The dot blot was used to confirm the total m^6^A levels in the TMZ sensitive cells and the resistant U87-MG/U251 cells. *P < 0.05, **P < 0.01, and ***P< 0.001 *versus* normal U87-MG/U251 cells.

Previously, elevated METTL3 levels have been associated with malignant characteristics of cancer cells (21), but its role in TMZ resistance in GBM has not been fully understood. Here, upon comparing the METTL3 levels between the TMZ-sensitive cells and the resistant cells, we found that the mRNA level of METTL3 was significantly higher (about 4.78-fold in U87-MG-TMZ and 4.48-fold in U251-TMZ) in the TMZ-resistant group than in the sensitive group (**Figure 1A**), which was further confirmed by western blot analysis (**Figure 1B**). We then examined m^6^A levels in the total RNAs from TMZ-sensitive cells and resistant cells using the colorimetric m^6^A quantification strategy, revealing significantly increased m^6^A levels in TMZ-resistant cells (**Figure 1C**) compared with TMZ-sensitive cells (approximately 4.16-fold in U87-MG-TMZ and 5.92-fold in U251-TMZ), confirmed by dot blot analysis (**Figure 1D**). These results suggest that METTL3 mediated m^6^A may contribute to TMZ-resistant GBM cells.

### METTL3 Contributes to the TMZ Resistance in GBM Cells

**Figure 2:**
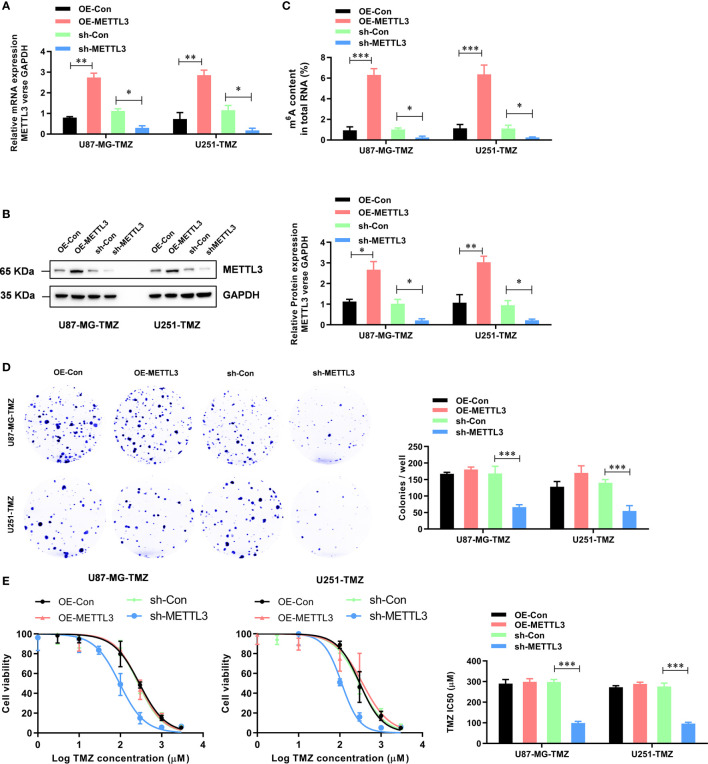
METTL3 contributes to the TMZ resistance in GBM cells. **(A)** The efficiency of overexpression and knockdown on the mRNA levels of METTL3 were analyzed by qRT-PCR. **(B)** The efficiency of overexpression and knockdown on the protein levels of METTL3 were analyzed by western blot. **(C)** The effect of METTL3 overexpression and knockdown on the total m^6^A RNA level was analyzed by the colorimetric m^6^A quantification assay. **(D)** The effect of METTL3 overexpression and knockdown on the cell proliferation was analyzed by the colony formation assay. **(E)** The effect of METTL3 overexpression and knockdown on the sensitivity to TMZ was analyzed by CCK-8 assay. *P < 0.05, **P < 0.01, and ***P < 0.001 *versus* indicated control U87-MG/U251 cells.

To further study the functional role of METTL3 in the regulation of TMZ resistance, we established METTL3-stable overexpression and knockdown U87-MG-TMZ and U251-TMZ cell lines. The efficiency of overexpression and knockdown on the mRNA and protein levels of METTL3 was verified by qRT-PCR (**Figure 2A**) and western blot (**Figure 2B**), respectively. Consistently, the m^6^A levels were significantly increased in METTL3 overexpressed U87-MG-TMZ and U251-TMZ cells, whereas decreased in METTL3 knockdown U87-MG-TMZ and U251-TMZ cells (**Figure 2C**). Compared with parental control, METTL3 knockdown GBM cells had a significantly lower ability to form colonies (**Figure 2D**), while TMZ-Resistant cells overexpressing METTL3 had no effect (**Figure 2D**). More importantly, METTL3 level was positively correlated with TMZ sensitivity. When METTL3 was knocked down, the IC_50_ value decreased from approximately 268.9 μM to 95.6 μM in U87-MG-TMZ cells and 296.0 μM to 110.6 μM in U251-TMZ, whereas the IC_50_ value remained unchanged in METTL3 overexpressing cells (**Figure 2E**). These results suggest that METTL3 silencing caused TMZ-resistant cells more sensitive to TMZ.

### METTL3 Contributes to the TMZ Resistance *via* m^6^A Modification

**Figure 3:**
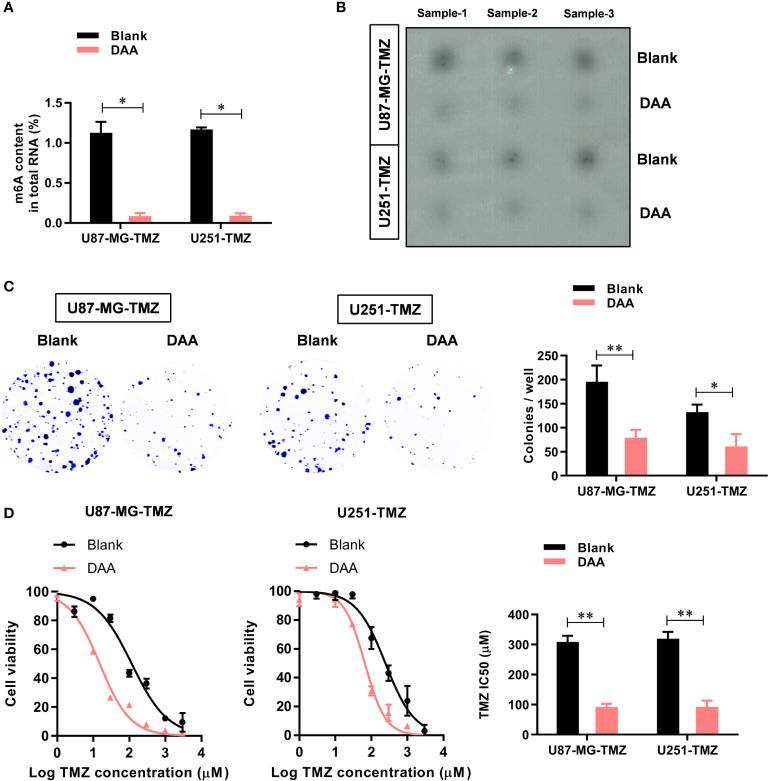
METTL3-mediated m^6^A modification contributes to the TMZ resistance. **(A)** The colorimetric m^6^A quantification assay was used to examine the total m^6^A levels in the control or DAA-treated U87-MG/U251-TMZ cells. **(B)** The dot blot was used to confirm the total m^6^A levels in the control or DAA-treated U87-MG/U251-TMZ cells. **(C)** The effect of DAA treatment on the cell proliferation was analyzed by the colony formation assay. **(D)** The effect of DAA treatment on the sensitivity to TMZ was analyzed by CCK-8 assay. *P < 0.05 and **P < 0.01 *versus* blank U87-MG/U251 cells.

**Figure 4:**
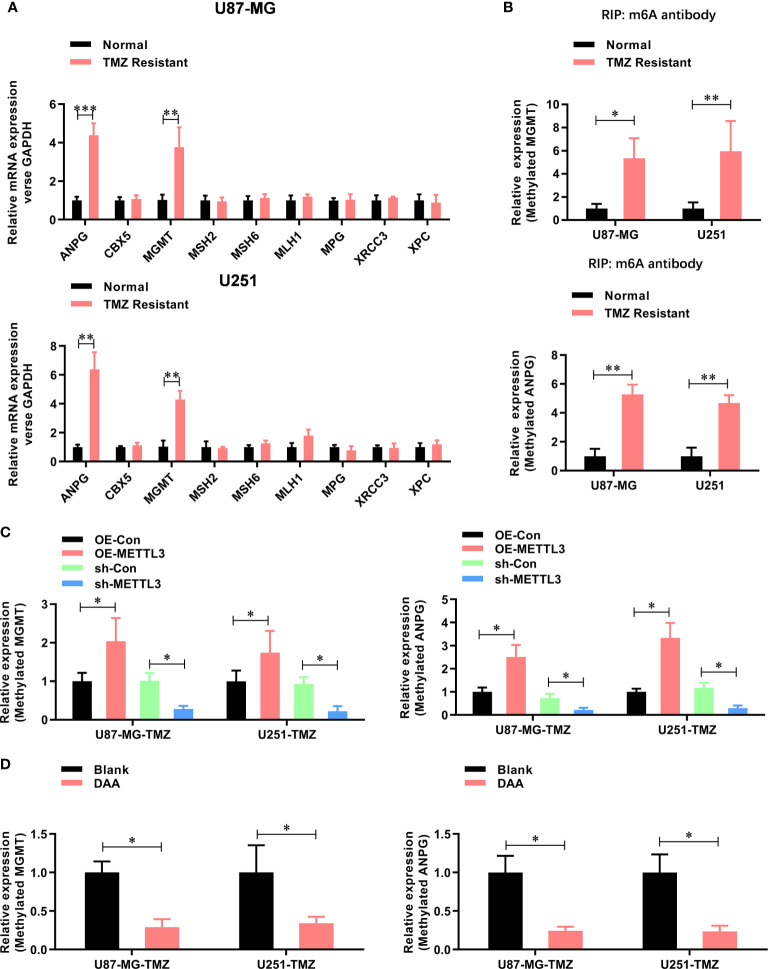
METTL3 contributes to the TMZ resistance *via* m^6^A modification of MGMT and ANPG mRNAs. **(A)** The expression mRNA level of TMZ resistant genes (ANPG, CBX5, MGMT, MSH2, MSH6, MLH1, MPG, XRCC3, and XPC) in normal and TMZ-resistant U87-MG/U251 cells were analyzed by real-time PCR. **(B)** The m^6^A-methylated level of MGMT and ANPG in normal and TMZ resistant U87-MG/U251 cells were analyzed by Me-RIP-real-time PCR. **(C)** The effect of METTL3 overexpression and knockdown on the m^6^A methylated level of MGMT and ANPG were analyzed by Me-RIP-real-time PCR. **(D)** The effect of DAA treatment on the m^6^A-methylated level of MGMT and ANPG were analyzed by Me-RIP-real-time PCR. *P < 0.05 and **P < 0.01 *versus* indicated control U87-MG/U251 cells.

To further study the functional role of METTL3-mediated m^6^A modification in the regulation of TMZ resistance, we inhibited methylation with a methylation inhibitor, 3-deazaadenosine (DAA, 100 µM). Consistent with our hypothesis, treating U87-MG/U251-TMZ cells with DAA led to a remarkable reduction in total m^6^A level (**Figure 3A**), which was verified by dot blot (**Figure 3B**). Moreover, compared with the parental control, DAA-treated GBM cells had a significantly lower ability to form colonies (**Figure 3C**). Furthermore, the IC_50_ value decreased from approximately 275.4 μM to 98.6 μM in U87-MG-TMZ cells and 288.2 μM to 108.3 μM in U251-TMZ (**Figure 3D**). The major repair enzymes, O6-methylguanine–DNA methyltransferase (MGMT) and alkylpurine–DNA–N-glycosylase (APNG), repairs the most cytotoxic lesions generated by TMZ. To analyze the underlying mechanism of METTL3-mediated m^6^A modification in the regulation of TMZ resistance, we screened a series of TMZ-resistant genes (ANPG, CBX5, MGMT, MSH2, MSH6, MLH1, MPG, XRCC3, and XPC), revealing that METTL3 overexpression significantly increased the MGMT and ANPG expression in GBM cells (**Figure 4A**).

Furthermore, the m^6^A methylation level (**Figure 4B**) of MGMT and ANPG were significantly increased in TMZ-resistant GBM cells. Notably, the m^6^A methylation level (**Figure 4C**) of MGMT and ANPG was significantly increased by METTL3 overexpression, which decreased by METTL3 knockdown or DAA treatment (**Figure 4D**). Collectively, these results demonstrate that METTL3 contributes to TMZ resistance *via* m^6^A modification.

### METTL3-Mediated m^6^A Modification Contributes to the TMZ Resistance *In Vivo*

**Figure 5:**
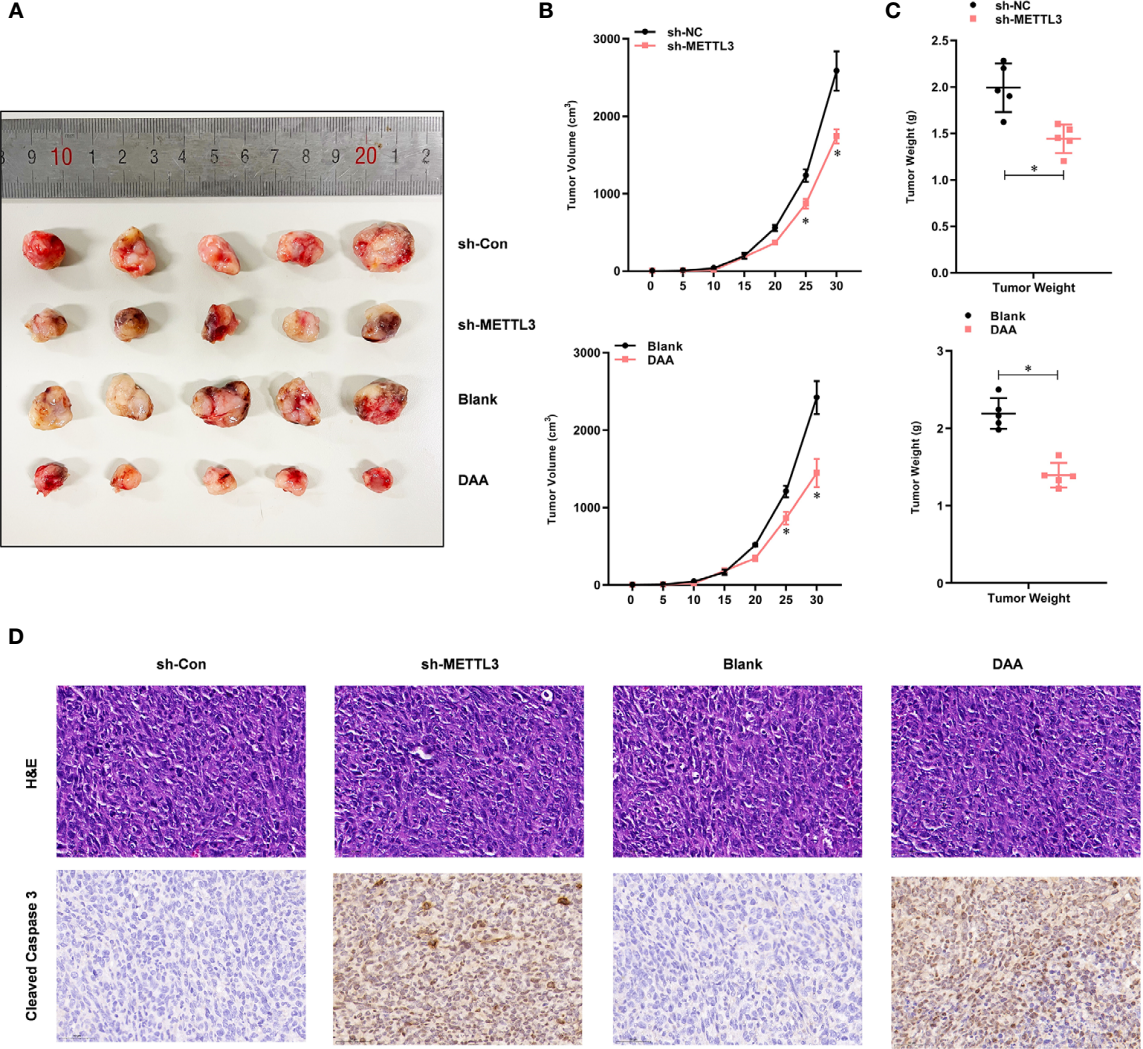
METTL3-mediated m^6^A modification contributes to the TMZ resistance in *vivo*. **(A)** Representative images of four groups of U87-MG-TMZ cells (shNC, shMETTL3, blank, and DAA)-derived subcutaneous tumors in the presence of TMZ. **(B)** Growth curve of tumor xenografts originated from four groups of U87-MG-TMZ cells (shNC, shMETTL3, blank, and DAA) in the presence of TMZ, **(C)** Weight of tumor xenografts originated from four groups of U87-MG-TMZ cells (shNC, shMETTL3, blank, and DAA) in the absence of TMZ. **(D)** IHC analysis of cleaved caspase-3 in tumor xenografts originated from four groups of U87-MG-TMZ cells (shNC, shMETTL3, blank, and DAA) in the presence of TMZ. *P < 0.05 *versus* indicated control U87-MG/U251 cells.

To investigate whether METTL3-mediated m^6^A modification was TMZ-resistant *in vivo*, we subcutaneously injected shMETTL3 or shNC-expressing U87-MG-TMZ cells into BALB/c NOD mice. After confirmation of GBM implantation, mice were treated with TMZ (66 mg/kg/d, 5 d per week, for 3 cycles). The tumor volume (**Figures 5A, B**) and weight (**Figure 5C**) of mice injected with shMETTL3 were significantly lower than those of xenografts expressing shNC. In contrast, mice treated with DAA (50 mg/kg/d, 5 d per week, for 3 cycles) and TMZ also resulted in a smaller tumor volume (**Figures 5A, B**) and weight (**Figure 5C**) than the blank group. IHC staining was performed to verify the expression of cleaved caspase-3. TMZ-treated xenografts with shMETTL3 expressing or DAA treatment had significantly increased level of cleaved caspase-3 compared with shNC or blank xenografts (**Figure 5D**). Taken together, these results demonstrate that METTL3-mediated m^6^A modification contributes to TMZ resistance *in vivo*.

## Discussion

GBM is one of the most aggressive types of cancer, for which no effective way of treatment is available (1). Despite advances in the development of chemotherapeutic agents, including targeted therapies, the overall survival after diagnosis is usually less than two years (26, 27). Recent experimental and clinical studies have shown that epigenetic regulation of GBM also plays an important role in promoting tumorigenesis and the development of drug resistance (2, 28, 29). m^6^A RNA methylation is an important RNA modification that has been shown to play an important role in the genesis and development of glioblastoma (30). In this study, we investigated the potential role of m^6^A methylation modification in the regulation of TMZ resistance and the feasibility of using the M^6^A inhibitor DAA as a therapeutic candidate.

In this study, we first analyzed the level of m^6^A RNA methylation in TMZ-sensitive and TMZ-resistant GBM cells and critical role of a major m^6^A methyltransferase METTL3 in TMZ resistance. METTL3 is an effective therapeutic target for various cancers, including pancreatic cancer (31), melanoma (32), colorectal cancer (33), and lung adenocarcinoma (16). METTL3 is overexpressed in hepatic cancer cells (HCC), and is associated with poor prognosis (34). METTL3 is highly expressed in ovarian cancer, significantly correlating with ovarian cancer grade, PT status, PN/PM status, and FIGO staging (35). These studies suggest that METTL3 is a potential oncogene. METTL3 enhances the m^6^A methylation by improving the stability of SOX2 in GBM, thereby promoting the stemness of glioma stem cells (GSCs) (25). Controversially, another team found that downregulation of METTL3 significantly promoted GSC self-renewal and tumorigenesis (36). In addition, ALKBH5 reduces m^6^A modification in GSCs and plays an important role in tumorigenesis in the progression of GBM by regulating FOXM1 expression (37). These findings highlight the importance of modifying m^6^A methylation in GBM progression. However, its role in TMZ resistance in GBM remains unclear. We found no significant difference in the METTL3 expression between normal and GBM tissues, and no association was observed between its expression level and the prognosis in GBM patients (GEPIA, data not shown). However, its expression is significantly elevated in TMZ-resistant GBM cells, compared to its parent TMZ-sensitive cells. Moreover, we verified the critical role of METTL3-mediated m^6^A modification in TMZ resistance in GBM cells. Both METTL3 silencing or total methylation inhibition with DAA increased the sensitivity of GBM cells to TMZ *in vitro* and *in vivo*. Meanwhile, we discovered that METTL3 overexpression dramatically increased the m^6^A methylation of MGMT and APNG, but did not affect the level of METTL14 (**Supplementary Figure 2**). However, METTL3 overexpression showed no effect on the colony formation of TMZ-resistant GBM cells, suggesting that a highly expressed and super-functional role of METTL3 in TMZ-resistant GBM cells, thus further overexpression of METTLE3 increased the total m^6^A methylated mRNAs, but did not enhance the cell proliferation ability of TMZ-resistant GBM cells.

Considering the molecular mechanism underlying the resistance of glioma cells to TMZ, a DNA alkylation agent, is currently the only chemotherapeutic drug having some efficacy against GBM, accompanied by surgery and radiation therapy (28). In *in vitro* and animal models, TMZ resistance can be mediated by MGMT, a DNA repair protein that removes the methyl group produced by TMZ from the O6 site of guanine, which represents the most cytotoxic damage (13, 38). GBM patients with methylated MGMT promoter had an increased overall survival compared with radiotherapy alone, and responded better in combination with TMZ and radiotherapy (14). However, 50% of GBM patients with MGMT methylation promoters do not survive for 2 years, and therefore receive only moderate benefits from TMZ treatment, suggesting additional resistance factors. Similarly, GBM patients with unmethylated MGMT also showed some response to TMZ, strongly suggesting that MGMT promoter methylation was not the only predictor of response to TMZ (39). In this study, we demonstrated that the expression of MGMT mRNA is also regulated by METTL3-mediated m^6^A modification, which contributes to TMZ resistance. Moreover, our investigation into other DNA repair modulating systems, including GATA4-mediated TMZ sensitivity (40), showed increased levels of APNG in METTL3 over-expressed GBM cells. The m^6^A or total mRNA levels of MGMT and APNG were elevated by METTL3 overexpression, whereas decreased by METTL3-silencing or DAA treatment. In summary, we have demonstrated that METTL3 promotes the TMZ resistance of glioma cells by increasing MGMT and ANPG in an m^6^A-dependent manner.

## Data Availability Statement

The original contributions presented in the study are included in the article/**Supplementary Material**. Further inquiries can be directed to the corresponding author.

## Ethics Statement

The animal study was reviewed and approved by The Ethics Committee of The Second Affiliated Hospital of Soochow University.

## Author Contributions 

JS and GC were major contributors to the molecular experiments and manuscript writing. XD and HL performed statistical analyses of the experimental data. SL and SC cultured the cells. YL and LW performed animal experiments. JY and ZQ reviewed the manuscript. JD designed and supervised the experiments. All authors contributed to the article and approved the submitted version.

## Funding

1. The Natural Science Foundation of Jiangsu Province (grant no. BK20201172). 2. Clinical Special Disease Diagnosis and Treatment Technology in Suzhou (grant no. LCZX201807). 3. Key project of the Jiangsu Health Commission (ZDB2020016). 4. Young Talent Development Plan of Changzhou Health Commission (grant no. 2020-233-CZQM2020013).

## Conflict of Interest

The authors declare that the research was conducted in the absence of any commercial or financial relationships that could be construed as a potential conflict of interest.

## Supplementary Material

Supplementary Figure 1 Generation of TMZ-resistant U87-MG and U251 cell lines. TMZ-resistant cell lines were generated by exposure of U87-MG and U251 cells to 200 μM of TMZ for over 6 months. The derived resistant cell lines were designated as U87-MG-TMZ resistant and U251-TMZ resistant, respectively. The cell survival ratio and half maximal inhibitory concentration (IC_50_) of TMZ for U87-MG and U251 was evaluated by CCK-8 assay.

Supplementary Figure 2 METTL3 overexpression or knockdown did not affect the level of METTL14 in the TMZ-resistance GBM cells. **(A)** The mRNA level of METTL14 in the METTL3 overexpression or knockdown TMZ-resistance GBM cells were analyzed by qRT-PCR. **(B)** The protein level of METTL14 in the METTL3 overexpression or knockdown TMZ-resistance GBM cells were analyzed by western blot.
