# Supplementary figures and images for: METTL3 Promotes the Resistance of Glioma to Temozolomide via Increasing MGMT and ANPG in a m6A Dependent Manner
Source: Front Oncol. 2021 Jul 15;11:702983. doi: 10.3389/fonc.2021.702983 (PMC8320395; doi:10.3389/fonc.2021.702983)

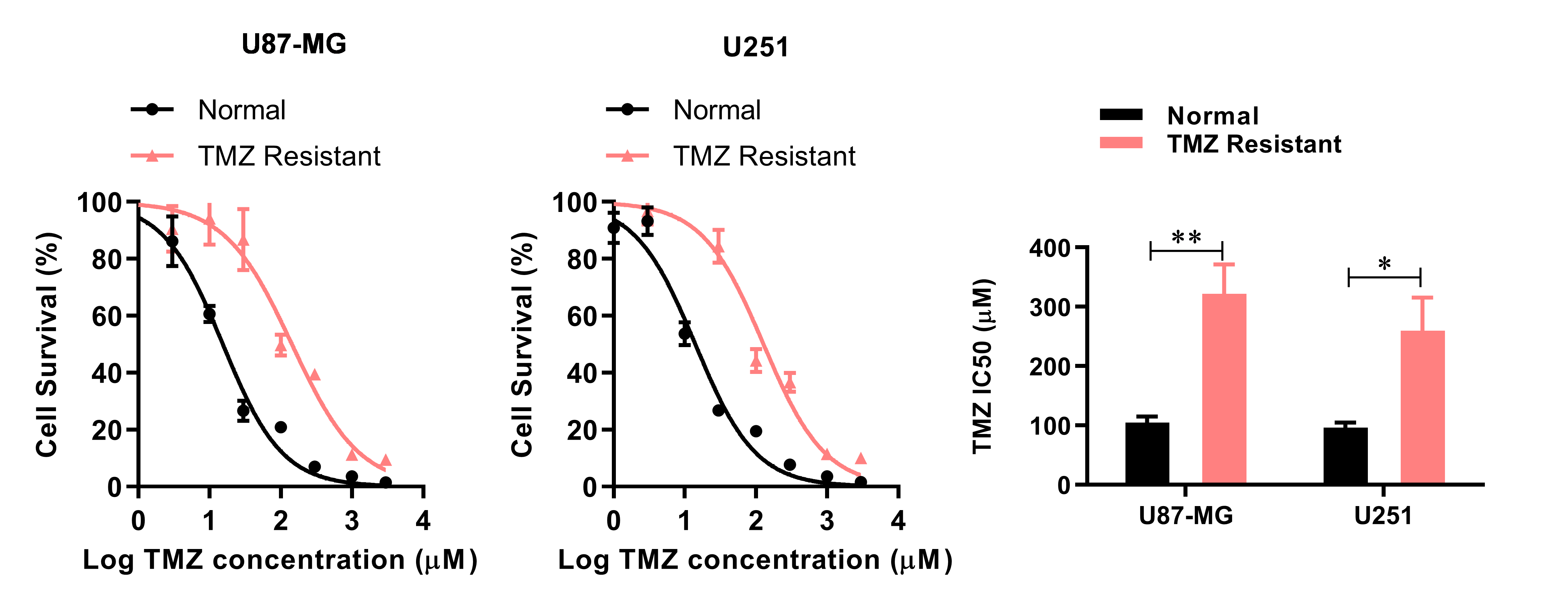

Supplement: Supplementary Figure 1 — Generation of TMZ-resistant U87-MG and U251 cell lines. TMZ-resistant cell lines were generated by exposure of U87-MG and U251 cells to 200 μM of TMZ for over 6 months. The derived resistant cell lines were designated as U87-MG-TMZ resistant and U251-TMZ resistant, respectively. The cell survival ratio and half maximal inhibitory concentration (IC50) of TMZ for U87-MG and U251 was evaluated by CCK-8 assay. [file Image_1.tif]

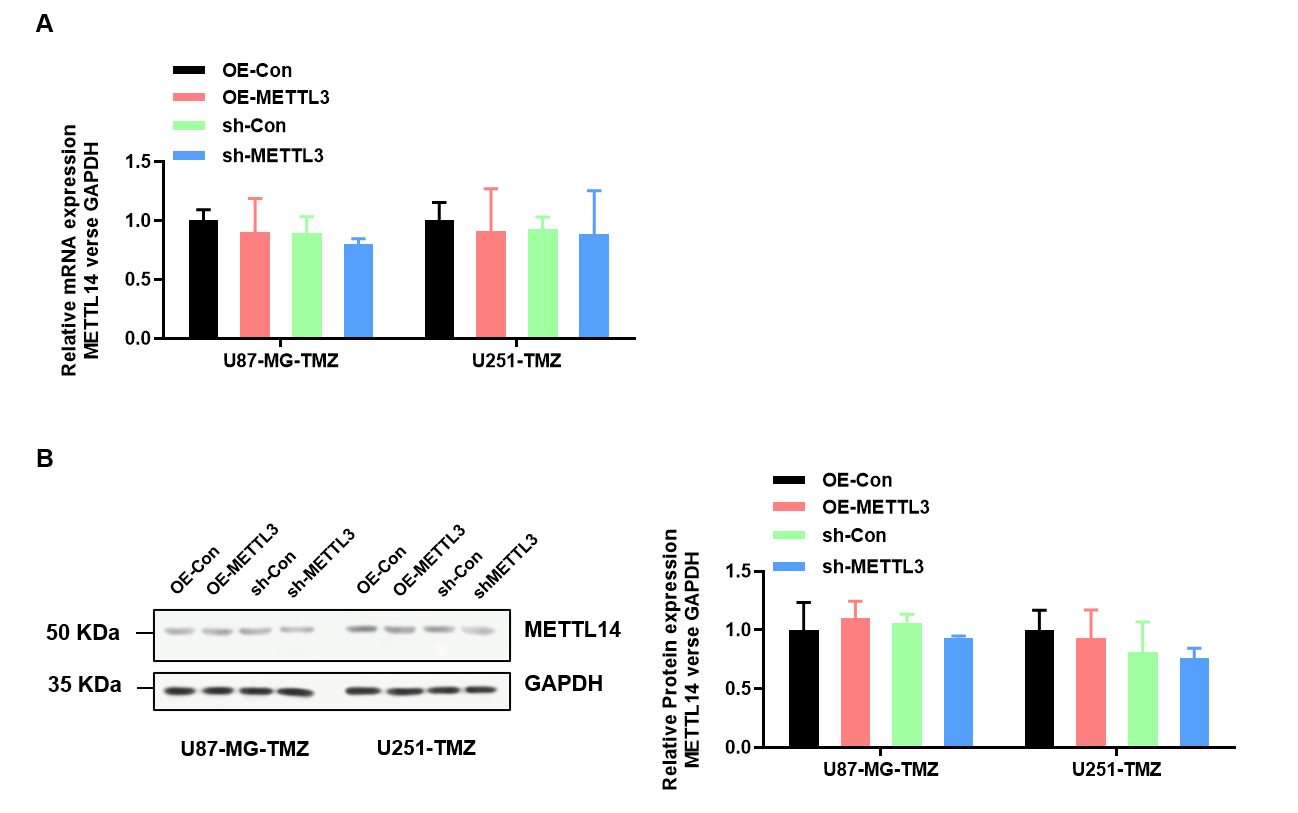

Supplement: Supplementary Figure 2 — METTL3 overexpression or knockdown did not affect the level of METTL14 in the TMZ-resistance GBM cells. (A) The mRNA level of METTL14 in the METTL3 overexpression or knockdown TMZ-resistance GBM cells were analyzed by qRT-PCR. (B) The protein level of METTL14 in the METTL3 overexpression or knockdown TMZ-resistance GBM cells were analyzed by western blot. [file Image_2.tif]
